# Temporal Transcriptomic and Metabolomic Reprogramming Unveils a Two-Phase Salt Tolerance Mechanism in *Apocynum venetum*

**DOI:** 10.3390/ijms27041917

**Published:** 2026-02-17

**Authors:** Syeda Wajeeha Gillani, Meng Wang, Lu Wang, Xueli Lu, Yu Bai, Yiru Song, Chen Meng, Xi Jia, Yiqiang Li, Chengsheng Zhang, Zongchang Xu

**Affiliations:** 1 Marine Agriculture Research Center, Tobacco Research Institute of Chinese Academy of Agricultural Sciences, National Center of Technology Innovation for Comprehensive Utilization of Saline-Alkali Land, Dongying 257300, China; gillani@caas.cn (S.W.G.); luxl100@163.com (X.L.); mengchen01@caas.cn (C.M.); liyiqiang@caas.cn (Y.L.); zhangchengsheng@caas.cn (C.Z.); 2 Marine Agriculture Research Center, Tobacco Research Institute of Chinese Academy of Agricultural Sciences, Qingdao Key Laboratory of Coastal Saline-Alkali Land Resources Mining and Biological Breeding, Qingdao 266100, China; 3 College of Agronomy, Qingdao Agricultural University, Qingdao 266109, China; wangmeng@qau.edu.cn (M.W.); bmyl1009@163.com (Y.B.); yirusong2024@163.com (Y.S.); 4 Yellow River Delta Modern Agriculture Research Institute, Shandong Academy of Agricultural Sciences, Dongying 257091, China; 18660191518@163.com (L.W.); jiaxi@shandong.cn (X.J.)

**Keywords:** NaCl stress, phenylpropanoids, salt-tolerant, transcriptomics, metabolomics, pioneer species

## Abstract

Soil salinization poses a major constraint to global agriculture. *Apocynum venetum*, a salt-tolerant halophyte, provides an effective model for investigating salt-adaptive strategies; however, the temporal dynamics of its tolerance-associated genes and metabolites remain unclear. In this study, integrated transcriptomics, metabolomics (UHPLC-MS), physiological assays, and weighted gene co-expression network analysis (WGCNA) were conducted to characterize early (7-day) and late (18-day) responses to 200 mM NaCl stress. NaCl stress significantly reduced chlorophyll content while increasing Na^+^ accumulation, MDA levels, antioxidant enzyme activities (SOD and CAT), and total flavonoid content. Early responses (NaCl7) were marked by accumulation of ferulic acid, rhamnetin, and 3,4-dihydrocoumarin, with activation of plant hormone (ABA, auxin, zeatin) and MAPK signaling pathways. Late responses (NaCl18) exhibited increased accumulation of scopoletin, formononetin, and caffeyl-alcohol, with enrichment of phenylpropanoid biosynthesis, glutathione metabolism, and photosynthesis-related pathways. WGCNA identified early-response hub genes, including *AOC*, *MAPKKK17/18*, *CYP98A*, and *CCoAOMT*, coordinating stress signaling and antioxidant metabolism. Late stress responses involved genes like *CPK*, *GST*, *CYCD3*, and *ARF*, modulating calcium signaling and ROS detoxification. Genes shared across phases included *CYP90C1*, *HD-ZIP*, *HSP20*, and *PP2C*, regulating protein stabilization and stress signaling. These findings reveal a two-phase salt tolerance strategy in *A. venetum*, integrating early signaling and late metabolic adaptation.

## 1. Introduction

*Apocynum venetum* L. is a perennial semi-shrub halophyte widely distributed across Southwest Asia and Europe, as well as the semi-arid, arid, and saline-alkaline regions of northern China, where it exhibits exceptional tolerance to drought and salinity stress [1,2,3]. Its adaptation to saline and sandy habitats relies on coordinated physiological and anatomical adaptations, including extensive root systems, osmotic adjustment, enhanced flavonoid biosynthesis, and Na^+^/K^+^ homeostasis [4,5,6,7,8]. Despite evidence that moderate salinity promotes flavonoid accumulation, the regulatory networks underlying salt tolerance remain poorly resolved due to limited transcriptomic data [9,10,11].

Soil salinization is one of the most severe abiotic constraints limiting agricultural productivity worldwide, substantially reducing arable land area, crop yield, and food security [12,13]. Currently, salinity affects approximately 20% of total cultivated land and over one-third of irrigated farmland, with affected areas expanding annually due to climate change, improper irrigation, and soil mismanagement [12]. In China, it is an expanding constraint, affecting more than 36 million hectares, particularly in northeastern, northwestern, and coastal regions [14,15]. Sodium chloride (NaCl) is a primary contributor to salinity in many of these landscapes, perturbing water potential, causing ion toxicity, and disrupting redox balance in sensitive crops [16,17]. Consequently, controlled NaCl exposure is widely used as a reproducible system to dissect salt-stress responses at physiological, transcriptomic, and metabolomic scales [16]. High soil salinity disrupts plant water uptake by lowering soil water potential, induces ionic toxicity through excessive Na^+^ and Cl^−^ accumulation, and promotes oxidative stress via excessive reactive oxygen species (ROS) generation, ultimately impairing growth and productivity [13,18,19]. Consequently, understanding plant responses to salinity stress is critical for sustaining agricultural systems and rehabilitating salt-affected lands [10,20].

Plants respond to salinity stress through coordinated physiological, biochemical, and molecular adjustments that mitigate osmotic stress, ion toxicity, and oxidative damage [18,21]. Early responses primarily involve osmotic adjustment, ion transport regulation, and stress signaling mediated by Ca^2+^, MAPK cascades, and phytohormones, enabling rapid stress perception and cellular protection [22,23]. Prolonged exposure triggers metabolic reprogramming, including enhanced antioxidant enzyme activity, accumulation of compatible solutes, and increased synthesis of secondary metabolites such as flavonoids and phenylpropanoids that stabilize membranes and scavenge ROS [13,24,25]. Halophytes exhibit particularly robust tolerance strategies by integrating transcriptional regulation with sustained metabolic acclimation, allowing survival and completion of their life cycle under high salinity conditions [16,20].

Previous studies have established *A. venetum* as a robust halophyte with strong physiological and molecular plasticity under salinity stress, making it a valuable system for dissecting salt tolerance mechanisms [26,27,28]. Transcriptomic and metabolomic analyses revealed activation of flavonoid and phenylpropanoid biosynthesis, hormone signaling, and stress-related regulatory networks in response to NaCl exposure, highlighting the importance of secondary metabolism in tolerance acquisition [26,29]. Physiological investigations further demonstrated enhanced antioxidant enzyme activities, osmolyte accumulation, SOS-mediated ion homeostasis, and induction of heat shock proteins and transcription factors during salt stress [6,30,31]. Comparative studies across halophytes and glycophytes indicate that salt stress responses are stage- and species-dependent, involving dynamic regulation of seed germination, redox balance, osmotic adjustment, and stress-responsive genes such as *DREB*, *WRKY*, *NAC*, and transporter families [8,32,33]. Functional validation studies showed that *A. venetum* genes related to flavonoid biosynthesis (*AvF3H*, *AvFLS*, and *AvF3GT*) and RNA helicase activity (*AvDH1*) significantly enhanced salt tolerance when overexpressed in heterologous systems, confirming their direct contribution to stress resilience [2,30,34,35].

Despite its recognized ecological and agronomic importance, *A. venetum* lacks a systematic, time-resolved multi-omics framework that distinguishes early and prolonged responses to salinity stress. In particular, how regulatory networks and metabolite profiles transition from rapid signaling to sustained metabolic acclimation remains unclear. To address this gap, the present study integrates physiological measurements with transcriptomic and widely targeted metabolomic analyses at two biologically meaningful stages of NaCl exposure: an early response phase (7 days) and a later acclimation phase (18 days). The objectives were to (i) characterize temporal changes in growth, ion regulation, and antioxidant physiology under NaCl stress; (ii) identify differentially expressed genes (DEGs) and co-expression modules associated with early versus late responses; and (iii) correlate time-specific transcriptomic patterns with metabolite shifts, with a particular focus on stress-mediated metabolic pathways. This temporal multi-omics framework provides mechanistic insight into salt tolerance in *A. venetum* and supports the development of strategies to improve its cultivation on saline-alkaline soils.

## 2. Results

### 2.1. Physiological Responses of A. venetum Seedlings Under NaCl Stress

**Figure 1:**
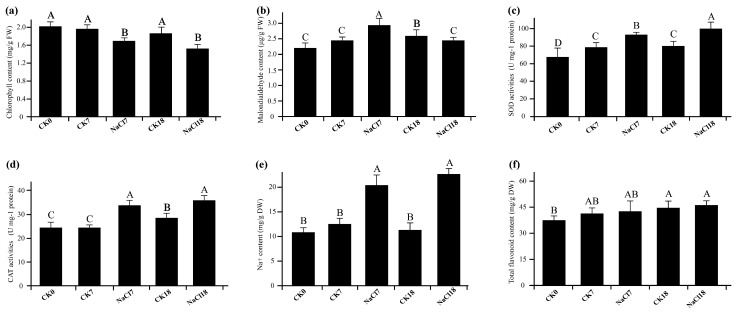
Physiological and biochemical responses of *A. venetum* to NaCl stress. (**a**) Chlorophyll content, (**b**) MDA, (**c**) SOD, (**d**) CAT activity, (**e**) Na^+^ content, and (**f**) total flavonoid content in leaves under control (CK0, CK7, and CK18), and 200 mM NaCl treatment (NaCl7 and NaCl18). Time points: day 0, 7, and 18. CK0: pre-treatment control (day 0), CK7 and CK18: time-matched controls for NaCl7 and NaCl18, respectively. Values: means ± SE (*n* = 3). Different capital letters indicate significant differences (*p* < 0.05).

Physiological and biochemical responses of *A. venetum* seedlings were evaluated under early (NaCl7) and late (NaCl18) NaCl stress conditions (Figure 1). Total chlorophyll content was significantly reduced in NaCl-treated plants (NaCl7 and NaCl18) compared with all control groups (CK0, CK7, and CK18) (*p* < 0.05; Figure 1a), while no significant differences were observed among control samples, indicating that chlorophyll decline was specifically induced by salt stress. Malondialdehyde (MDA) content was significantly elevated in NaCl7 relative to all other treatments (*p* < 0.05; Figure 1b), whereas NaCl18 exhibited a moderate but significant reduction compared with NaCl7, suggesting partial mitigation of membrane lipid peroxidation during a late stress response.

Antioxidant enzyme activities showed a stress- and time-dependent response. Sodium dismutase (SOD) activity increased significantly under NaCl treatment, with NaCl18 displaying the highest activity among all treatments (*p* < 0.05; Figure 1c), indicating enhanced ROS-scavenging capacity during the late stress phase. Catalase (CAT) activity was also significantly higher in NaCl-treated samples compared with controls (*p* < 0.05), but no significant difference was detected between NaCl7 and NaCl18 (Figure 1d), suggesting sustained H_2_O_2_ detoxification throughout stress progression. Na^+^ content increased markedly in both NaCl7 and NaCl18 compared with all control treatments (*p* < 0.05; Figure 1e). Although NaCl18 showed a slightly higher Na^+^ accumulation than NaCl7, the difference was insignificant, indicating comparable ionic loading under early and late stress conditions. Total flavonoid content exhibited a gradual increasing trend with stress duration (Figure 1f). NaCl18 showed significantly higher flavonoid levels than NaCl7 (*p* < 0.05), whereas NaCl7 and NaCl18 did not differ significantly from their respective controls (CK7 and CK18), suggesting that flavonoid accumulation becomes more pronounced during the late phase of NaCl stress.

### 2.2. Transcriptional Responses of A. venetum Seedlings Under NaCl Stress

**Figure 2:**
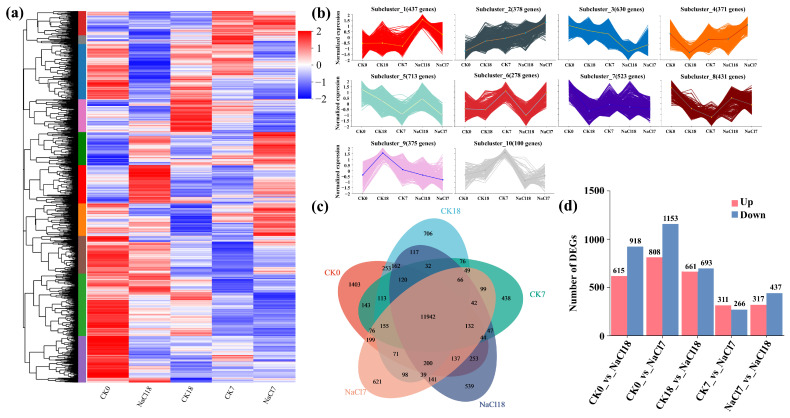
Transcriptomics profiling under control (CK) and 200 mM NaCl stress (NaCl). (**a**) Hierarchical clustering heatmap, (**b**) k-means clustering, (**c**) Venn diagram of samples, and (**d**) bar plot of differentially expressed genes (DEGs) across comparison groups under control (CK) and salt stress (NaCl). Time points: day 0, 7, and 18. CK0: pre-treatment control (day 0), CK7 and CK18: time-matched controls for NaCl7 and NaCl18, respectively. Red indicates upregulated genes, and blue represents downregulated genes.

Transcriptome sequencing of 15 tolerant samples (5 samples × 3 replicates) generated 109.82 Gb of high-quality clean data, with each sample yielding more than 5.63 Gb of clean reads and with Q30 values exceeding 94.83%, confirming robust sequencing quality. Hierarchical clustering of DEGs revealed clear segregation by treatment and time point, with CK0 clustering independently and NaCl-treated samples (NaCl7 and NaCl18) exhibiting expression profiles distinct from their respective controls, indicating salinity is the primary driver of transcriptomic variation (Figure 2a). K-means clustering classified DEGs into ten expression subclusters (Figure 2b). Subclusters 1, 5, 7, and 8 exhibited strong induction at NaCl18, implicating these genes in late response, whereas subclusters 3, 9, and 10 were progressively downregulated under NaCl stress. Several subclusters showed transient induction at NaCl7 followed by repression at NaCl18, consistent with early stress signaling and acclimation responses. Venn analysis identified 11,942 genes expressed across all treatments, representing core cellular functions (Figure 2c). NaCl7 displayed the highest number of treatment-specific DEGs (621), indicative of an early response, while NaCl18 contained 539 unique DEGs, reflecting extensive transcriptional remodeling during late response. Overall, NaCl stress predominantly induced gene downregulation, except at CK7 vs. NaCl7, where upregulated NaCl7 genes outnumbered downregulated genes (Figure 2d).

Differential comparison analysis revealed 311 DEGs in CK7 vs. NaCl7 and 661 upregulated DEGs in CK18 vs. NaCl18, suggesting activation of tolerance-related genes under late response. Principal component analysis (PCA) further supported treatment- and time-dependent transcriptional variation (Figure S1). The first two components explained 28.81% of total variation (PC1 = 16.25%, PC2 = 12.56%). PC1 separated NaCl-treated samples from controls, whereas PC2 distinguished 7-day and 18-day treatments. Tight clustering of biological replicates confirmed data reproducibility. Additionally, NaCl18 exhibited the greatest divergence from the rest of the samples, indicating maximal transcriptomic reprogramming under late response.

### 2.3. Transcriptional Regulation and Enriched Salt-Responsive Pathways

GO annotation of DEGs revealed significant enrichment in molecular functions related to binding and catalytic activity, cellular components associated with membranes and cell parts, and biological processes primarily involving metabolic and cellular functions (Figure S2). These enrichments were more pronounced in CK18 vs. NaCl18 than in CK7 vs. NaCl7, indicating gradual activation of tolerance-related processes under late response. KEGG classification assigned most DEGs to metabolism, genetic information processing, and environmental information processing pathways (Figure S3). Metabolic pathways, particularly those related to carbohydrate, amino acid, lipid, and secondary metabolite pathways, were highly represented, indicating broad metabolic adjustment under salinity. Pathways associated with environmental adaptation, signal transduction, and protein processing were predominantly annotated to CK18 vs. NaCl18, whereas transcription- and translation-related pathways were more prominent in NaCl7 vs. NaCl18, suggesting time-specific regulation of gene expression machinery.

KEGG enrichment analysis identified major stress-responsive pathways, including plant hormone signal transduction, MAPK signaling, phenylpropanoid and flavonoid biosynthesis, protein processing in the endoplasmic reticulum, and oxidative phosphorylation (Figure S4). Early responses (NaCl7) were characterized by enrichment of brassinosteroid and zeatin biosynthesis, whereas late responses (NaCl18) enriched photosynthesis antenna proteins and mannose-type glycan biosynthesis. Hormone signaling, MAPK pathways, and phenylpropanoid and glutathione metabolism were activated at both stages. Overall, an early response (7 days) primarily activated transient stress-response genes, while a late response (18 days) induced robust transcriptional reprogramming, including growth- and tolerance-related pathways, indicating a shift from rapid signaling to sustained stress adaptation.

### 2.4. Metabolomic Responses of A. venetum Seedlings Under NaCl Stress

**Figure 3:**
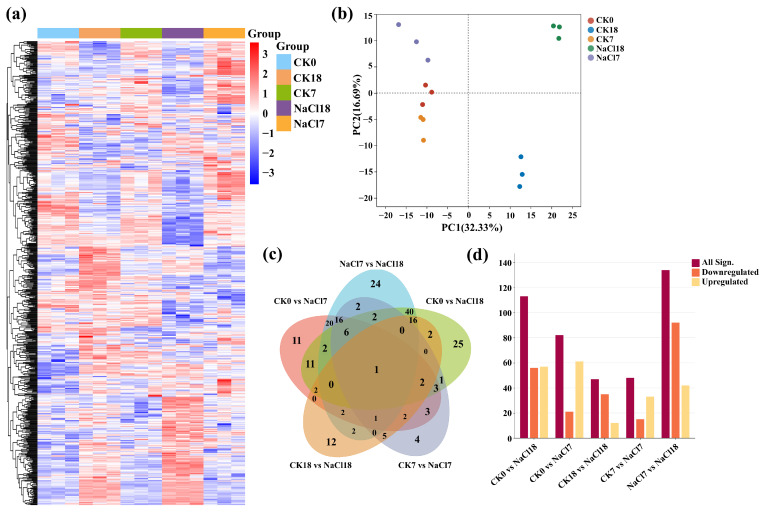
Metabolomic profiling under control (CK) and 200 mM NaCl stress (NaCl). (**a**) Hierarchical clustering heatmap, (**b**) PCA plot, (**c**) Venn diagram of sample overlaps, and (**d**) bar plot of differentially accumulated metabolites (DAMs) across comparison groups. Time points: 0 = day 0, 7 = day 7, 18 = day 18. Red indicates upregulated metabolites, and blue represents downregulated metabolites.

Untargeted metabolomic profiling corroborated transcriptomic findings, revealing dynamic, time-dependent metabolic reprogramming under NaCl stress (Figure 3).

**Figure 4:**
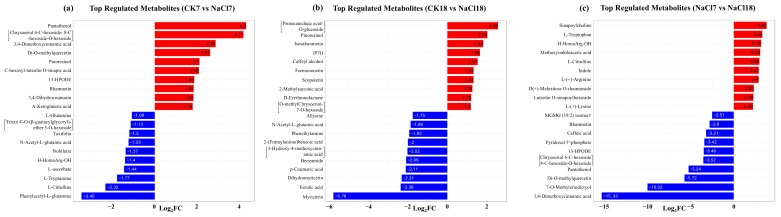
Bar plot showing log_2_ fold change (FC) of the most up- and downregulated DAMs in salt stress-related comparison groups. Control (CK) and salt stress (NaCl). Time points: 0 = day 0, 7 = day 7, 18 = day 18.

Heatmap clustering indicated distinct accumulation patterns across treatments, with NaCl18 exhibiting the most pronounced changes, partially resembling CK0 profiles (Figure 3a). PCA separated 18-day samples (CK18, NaCl18) from 7-day treatments, with PC1 explaining 32.33% and PC2 16.69% of the total variation (49.02%), indicating that a late stress response drives major metabolic shifts (Figure 3b). Biological replicates clustered tightly, confirming data robustness. Venn analysis revealed both shared and unique metabolites (Figure 3c). A single metabolite was common across all comparison groups, whereas CK7 vs. NaCl7 and CK18 vs. NaCl18 contained 4 and 12 unique metabolites, respectively, reflecting increasing metabolic divergence with stress duration. Notably, NaCl7 vs. NaCl18 had 24 unique metabolites, indicating time-dependent accumulation associated with early-to-late stress responses. Differential showed an early response (CK7 vs. NaCl7) with more upregulated metabolites, including flavonoids such as pantothenol (log_2_FC 4.3), di-methylquercetin (log_2_FC 2.61), rhamnetin (log_2_FC 1.83), and 3,4-dihydrocoumarin (log_2_FC 1.82), reflecting antioxidant activation (Figure 3d and Figure 4). A late response (CK18 vs. NaCl18) exhibited accumulation of protacatechuic acid-glucoside (log_2_FC 2.58) followed by pinoresinol (log_2_FC 2.04), iP7G (1.66), caffeyl-alcohol (1.54), formononetin (1.34), and scopoletin (1.34), indicating enhanced secondary metabolism. NaCl7 vs. NaCl18 showed increased amino acids and derivatives, including sinapoylcholine (log_2_FC 3.82), tryptophan (log_2_FC 3.32), citrulline (log_2_FC 2.99), indole (log_2_FC 2.91), arginine (log_2_FC 2.9), and lysine (log_2_FC 2.23), supporting osmotic adjustment and stress signaling, while non-essential metabolites such as caffeic acid, rhamnetin, and di-methylquercetin were selectively downregulated (Figure 4). These results demonstrate that early responses prioritize rapid antioxidants and flavonoid-mediated defense, whereas late responses orchestrate selective and sustained metabolic adjustments in secondary metabolism, amino acids, and osmoprotectants, underpinning long-term salt tolerance in tolerant *A. venetum*.

### 2.5. Metabolic Reprogramming and Enriched Salt-Responsive Pathways

KEGG enrichment analysis of DAMs identified several key salt-responsive pathways, including secondary metabolite biosynthesis (ko01110), phenylpropanoid biosynthesis (ko00940; ko01061), and arginine biosynthesis (ko00220) (Figure S5). Early responses (CK7 vs. NaCl7) were predominantly characterized by enrichment of arginine biosynthesis and taurine and hypotaurine metabolism (ko00430), with secondary metabolite biosynthesis showing the highest enrichment. In contrast, late responses (CK18 vs. NaCl18) exhibited enrichment of phenylpropanoid biosynthesis and D-alanine metabolism, alongside sustained activation of secondary metabolite pathways (Figure S5). While phenylpropanoid and secondary metabolite biosynthesis were consistently enriched at both time points, isoquinoline alkaloid biosynthesis (ko00950) was exclusively enriched in NaCl7 vs. NaCl18. Collectively, these results indicate that early and late stress responses drive coordinated metabolic and regulatory adjustments that underpin salt tolerance in *A. venetum*.

### 2.6. Integrated Transcriptome-Metabolome Responses Under NaCl Stress

**Figure 5:**
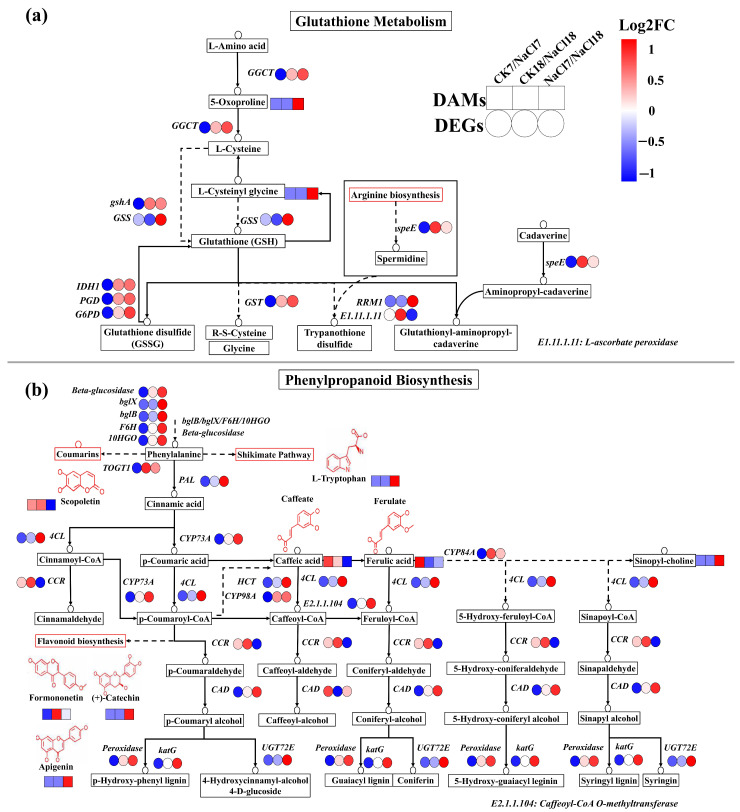
KEGG Pathway illustration showing the (**a**) glutathione metabolism and (**b**) phenylpropanoid biosynthesis pathways. Control (CK) and NaCl stress (NaCl) at time points: 0 = day 0, 7 = day 7, 18 = day 18.

Integrated transcriptomic and metabolomic analyses revealed clear temporal differentiation of NaCl-responsive pathways in *A. venetum* (Figure 5). Early responses (CK7 vs. NaCl7) were characterized by coordinated enrichment of plant hormone signal transduction (ko04075), MAPK signaling (ko04016), and phenylpropanoid biosynthesis (ko00940), arginine biosynthesis (ko00220), and glutathione metabolism (ko00480). In contrast, late responses (CK18 vs. NaCl18) were dominated by enrichment of phenylpropanoid biosynthesis (ko00940), photosynthesis-related pathways (ko00195), and glutathione metabolism (ko00480), indicating a shift from rapid signaling to metabolic acclimation. Phenylpropanoid biosynthesis and glutathione metabolism remained consistently enriched across all comparisons, including NaCl7 vs. NaCl18, and were therefore selected for detailed integrated analysis. For genes represented by multiple transcripts, expression values were summarized at the gene level by selecting the transcript with the highest mean expression across biological replicates. Transcript-level information is provided in Table S1.

Transcriptomic changes closely mirrored metabolite dynamics within the glutathione metabolism pathway (ko00480) (Figure 5a). Genes like GSS and RRM1 were exclusively upregulated in NaCl7 vs. NaCl18, despite reduced expression relative to controls (Figure 5a). Genes like *GSS* and *RRM1* were exclusively upregulated in NaCl7 vs. NaCl18, despite reduced expression relative to controls (Figure 5a). L-ascorbate peroxidase (*E1.11.1.11*) displayed the highest expression in CK18 vs. NaCl18, indicating its prominent role in late-stage oxidative stress mitigation. Additionally, antioxidant- and redox-related genes, including *gshA*, *GGCT*, *IDH1*, *PGD*, *G6PD*, *GST*, and *speE*, were suppressed during early stress (CK7 vs. NaCl7) but markedly induced in NaCl18-related comparisons, reflecting reinforcement of antioxidant capacity during late response. Consistently, glutathione-related metabolites such as 5-oxoproline and L-cysteinyl glycine were exclusively accumulated in NaCl7 vs. NaCl18 and elevated in NaCl18, indicating intensified redox buffering during late response.

Within the phenylpropanoid biosynthesis pathway, core structural genes, such as *Peroxidase*, *CAD*, *TOGT1*, and *CYP98A/84A/73A*, were notably induced in CK18 vs. NaCl18 and NaCl7 vs. NaCl18, indicating activation of phenylpropanoid metabolism during late responses (Figure 5b). In contrast, *PAL*, *HCT*, *4CL*, *bgLX*/*bglB*, *UGT72E*, and *F6H* were exclusively upregulated in NaCl7 vs. NaCl18, suggesting increased phenolic modification and glycosylation late response. Downregulation of *CCR* in NaCl7 vs. NaCl18, together with selective repression of other genes in CK7 vs. NaCl7, indicates a temporal shift towards stress-adaptive phenylpropanoid flux (Figure 5b). Metabolite profiling supported these trends, with scopoletin and caffeic acid upregulated at both early and late responses but downregulated in NaCl7 vs. NaCl18, while ferulic acid was exclusively induced in CK7 vs. NaCl7. Compounds including L-Tryptophan, sinapoyl-choline, (+) catechin, and apigenin accumulated predominantly in NaCl7 vs. NaCl18, whereas formononetin was uniquely upregulated in CK18 vs. NaCl18, underscoring functional specialization of phenylpropanoid derivatives over time. Collectively, these integrated analyses demonstrate a temporal progression from early signaling activation to sustained metabolic reprogramming in *A. venetum*, with phenylpropanoid biosynthesis and glutathione metabolism forming central hubs underlying both early defense and long-term NaCl tolerance.

### 2.7. WGCNA-Based Identification of Salt Tolerance-Related Gene Networks

**Figure 6:**
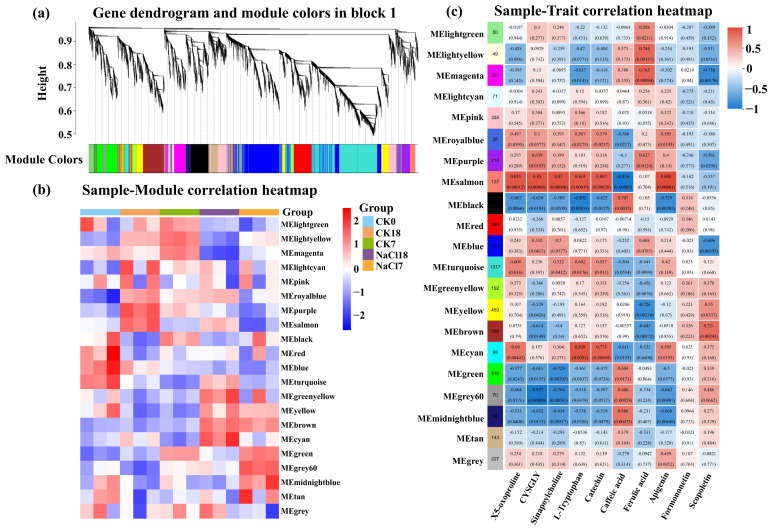
WGCNA analysis of DEGs. (**a**) Gene dendrogram with module color assignments. (**b**) Sample-module correlation heatmap. (**c**) Module-trait (metabolites) correlation heatmap. Control (CK) and NaCl stress (NaCl) at time points: 0 = day 0, 7 = day 7, 18 = day 18. CYSGLY: L-Cysteinyl glycine.

WGCNA identified 21 co-expression modules significantly associated with NaCl stress and KEGG-enriched metabolites (Figure 6a). Sample-module correlation showed that MEgrey60 and MEmidnightblue were exclusively and positively correlated with an early response (NaCl7; *p* < 0.05), whereas MEcyan and MEgreenyellow were specifically correlated with a late response (NaCl18; *p* < 0.05) (Figure 6b).

In addition, MEyellow and MEbrown showed strong positive correlations with both NaCl7 and NaCl18 samples compared with controls (CK7, CK18) (*p* < 0.05), central regulatory roles across salt-stress phases. Module-trait correlation heatmap revealed that MEgrey60 and MEmidnightblue were positively correlated with early-induced metabolites, including caffeic acid (*p* < 0.01) and scopoletin (Figure 6c). The MEcyan module exhibited strong positive correlation with L-tryptophan, catechin, 5-oxoproline (*p* < 0.001), and apigenin (*p* < 0.05), all enriched at NaCl18, highlighting its involvement in late metabolic acclimation. MEyellow and MEbrown were positively correlated with metabolites upregulated at both time points, such as scopoletin (*p* < 0.05) and formononetin, whereas MEgreenyellow showed weaker non-significant correlations.

**Figure 7:**
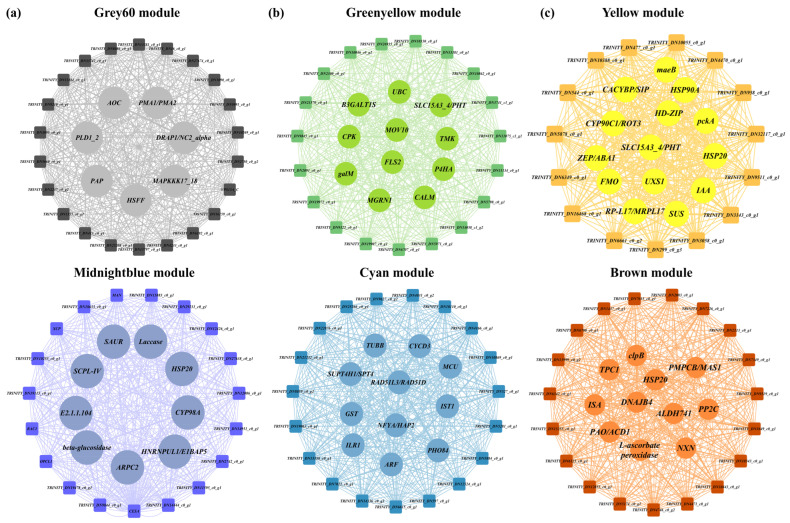
Top 30 hub genes associated with salt stress-mediated upregulated modules correlated with different samples in tolerant *A. venetum* plants. (**a**) Early stress-responsive (NaCl7) modules; (**b**) late stress-responsive (NaCl18) modules, (**c**) both early and late NaCl stress-responsive (NaCl7 and NaCl18) genes consistent in tolerance.

Hub-gene analysis revealed distinct temporal regulatory signatures (Figure 7a; Table S2). In NaCl7, key early-response hub genes included *AOC* (Allene oxide cyclase), *PMA1/PMA2* (H^+^-transporting ATPase), and *MAPKKK17_18* (Mitogen-activated protein kinase kinase kinase 17/18) in MEgrey60 and *HSP20* (Heat shock protein 20 family protein), *Laccase* (multicopper oxidase), and *CYP98A* (cytochrome P450 enzyme) in MEmidnightblue, indicating rapid activation of hormonal signaling, ion homeostasis, protein stabilization, and phenylpropanoid metabolism.

Under NaCl18, hub genes in MEcyan included cyclin D3 (*CYCD3*), auxin response factor (*ARF*), nuclear transcription factor Y subunit A (*NFYA*), glutathione S-transferase (*GST*), mitochondrial calcium uniporter (*MCU*), and phosphate transporter (*PHO84*) in MEcyan, indicating late tolerance mechanisms (Figure 7b; Table S2). MEgreenyellow harbored tolerance-associated genes such as flagellin-sensitive 2 (*FLS2*), calcium-dependent protein kinase (*CPK*), ubiquitin-conjugating enzyme (*UBC*), *MGRN1*, calmodulin (*CALM*), and GTP-binding protein (*GALM*), implicating calcium signaling and protein turnover in long-term adaptation (Figure 7b; Table S2). Genes consistently upregulated at both time-points included flavin-containing monooxygenase (*FMO*), cytochrome P450 90C1 (*CYP90C1*), homeodomain-leucine zipper (*HD-ZIP*), zeaxanthin epoxidase/ABA1 (*ZEP/ABA1*), heat shock protein 90A (*HSP90A*), indole-3-acetic acid (*IAA*), and sucrose synthase (*SUS*) (Figure 7c; Table S2), whereas MEbrown contained protein phosphatase 2C (*PP2C*), heat shock protein 20 (*HSP20*), ClpB protease (*clpB*), aldehyde dehydrogenase 741 (*ALDH741*), transcription factor 1 (*TC1*), isoamylase (*ISA*), and polyamine oxidase (*PAO*) (Figure 7c; Table S2), reflecting enhanced hormone signaling, antioxidant defense, and carbohydrate metabolism as a sustained tolerance response.

Collectively, these results demonstrate a temporally coordinated regulatory network in which early signaling and ion regulation transition into sustained metabolic, antioxidant, and phenylpropanoid-mediated tolerance in *A. venetum* under NaCl stress.

## 3. Discussion

In this study, integrated transcriptomic (RNA-seq), metabolomic (UHPLC-MS), and WGCNA analyses were employed to elucidate the molecular and metabolic basis of salt tolerance in tolerant *A. venetum* seedlings under NaCl stress. Comparative analyses at early (7 days; NaCl7) and late (18 days; NaCl18) stress responses revealed distinct yet interconnected adaptive programs, indicating a progressive salt-stress response in which rapid signal perception and hormonal regulation during early responses transition into sustained transcriptional and metabolic reprogramming under late responses.

The physiological and biochemical alterations observed under 200 mM NaCl are consistent with canonical salt-stress responses. Increased Na^+^ accumulation in NaCl7 and NaCl18 confirms substantial ionic loading under salinity, which can inhibit Ca^2+^ and K^+^ uptake, disrupt ion balance, and impair multiple metabolic pathways [36,37]. The significant decline in chlorophyll content at both NaCl7 and NaCl18 likely reflects salt-induced pigment degradation and inhibition of photosynthesis capacity under oxidative stress [38]. This indicates an early regulatory adjustment of photosynthesis in response to salinity. Salt tolerance is generally associated with coordinated changes in osmolyte accumulation, physiological traits, and antioxidant activities [8,39]. The pronounced increase in MDA at NaCl7 indicates severe early membrane lipid peroxidation, whereas its partial reduction at NaCl18 suggests progressive acclimation as antioxidant defenses become established during prolonged stress [8,40,41]. This pattern reflects a transition from acute stress injury to physiological stabilization. Concomitantly, the significant induction of SOD and CAT activities under NaCl supports enhanced ROS scavenging and maintenance of redox homeostasis during stress progression [38,42,43]. These responses underscore the central role of antioxidant systems in salt tolerance. Finally, the increase in total flavonoids, particularly at late stages, is consistent with their roles in antioxidant protection and redox buffering under salinity [44]. Collectively, these responses contribute to enhanced salt tolerance in *A. venetum*.

NaCl treatment induced extensive, time-dependent transcriptional remodeling, with hierarchical clustering and principal component analyses clearly separating NaCl18 samples from other treatments. Both early and late responses were characterized by strong enrichment of plant hormone signal transduction (ko04075), MAPK signaling (ko04016), and phenylpropanoid/flavonoid biosynthesis pathways, consistent with rapid stress sensing and hormone-mediated regulatory crosstalk essential for salt acclimation [45,46,47]. Early responses additionally showed enrichment of brassinosteroid and zeatin biosynthesis, suggesting a role for growth-regulating hormones in initial stress adjustment [47,48], whereas late responses were marked by enrichment of photosynthesis antenna proteins and mannose-type glycan biosynthesis, reflecting longer-term physiological adjustment, photosynthetic optimization, and cellular structural stabilization under sustained salinity [49,50]. The NaCl-induced reduction in chlorophyll content, together with the partial decline in MDA levels at the late response stage, is consistent with the late-stage enrichment of photosynthesis antenna protein pathways. This coordinated adjustment reflects effective photosynthesis and redox acclimation during late response to salinity.

Distinct temporal gene-expression subclusters further supported this progression. Genes induced at NaCl7 but repressed at NaCl18 likely represent transient early-response programs associated with rapid signaling [51], whereas subclusters strongly induced at NaCl18 underpin salt tolerance through sustained metabolic adjustment and stress protection [50]. Subclusters consistently upregulated at both stages represent core stress-responsive genes contributing to basal and prolonged tolerance [51], a pattern consistent with observations in other halophytes where early responses prioritize signaling and growth modulation, while late responses favor antioxidant defense, osmolyte accumulation, and membrane remodeling [52,53].

Integrated transcriptome-metabolome analyses revealed tight coordination between gene regulation and metabolite accumulation. Early hormone- and MAPK-mediated signaling transitioned to late metabolic acclimation dominated by phenylpropanoid biosynthesis, glutathione metabolism, and photosynthesis-related pathways [27,33]. Phenylpropanoid biosynthesis emerged as a central salt-responsive pathway, with late-stage upregulation of *PAL*, *HCT*, *4CL*, *CAD*, and glycosyltransferases (*UGT72E*), promoting the synthesis of flavonoids, lignin precursors, and polyphenols that enhance ROS scavenging, membrane stability, and cell wall reinforcement [54,55,56,57]. Early accumulation of ferulic acid reflected rapid antioxidant activation [58], whereas sustained modulation of scopoletin and caffeic acid indicated prolonged redox regulation [59,60,61]. Late accumulation of formononetin, apigenin, sinapoyl-choline, L-tryptophan, and catechin, together with increased arginine, lysine, and citrulline, highlights a metabolic shift toward long-term acclimation involving secondary metabolism, osmotic regulation, and polyamine/nitric oxide biosynthesis [62,63,64,65]. The progressive increase in total flavonoid content at NaCl18 aligns with transcriptional activation of phenylpropanoid biosynthesis genes and accumulation of flavonoid derivatives. Glutathione metabolism was prominently induced during the late response, reflecting reinforcement of redox homeostasis under prolonged stress [66]. Upregulation of *GSS*, *GST*, *IDH1*, *PGD*, *speE*, and L-ascorbate peroxidase, along with accumulation of cysteinyl-glycine and 5-oxoproline, indicates enhanced glutathione turnover and antioxidant capacity during chronic NaCl exposure [67,68].

WGCNA identified distinct co-expression modules and hub genes associated with NaCl stress responses in *A. venetum*. The MEgrey60 and MEmidnightblue modules were exclusively and positively correlated with the early response (NaCl7; *p* < 0.05), indicating their roles in rapid stress perception and signaling. Key early-response hub genes in MEgrey60 included *AOC*, *PMA1/PMA2*, and *MAPKKK17/18*, reflecting activation of jasmonate signaling, ion homeostasis, and MAPK-mediated signal transduction during salt shock [69,70,71]. In MEmidnightblue, hub genes such as *HSP20*, *laccase*, and *CYP98A* indicate early engagement of protein stabilization, redox regulation, and phenylpropanoid metabolism, supporting rapid tolerance initiation [72,73].

In contrast, the MEcyan and MEgreenyellow modules were specifically associated with the late response (NaCl18), highlighting their involvement in long-term tolerance mechanisms. Hub genes in MEcyan, including *CYCD3*, *ARF*, *NFYA/HAP2*, *GST*, and *SUPT4H1/SPT4*, suggest coordinated regulation of cell-cycle progression, hormone signaling, transcriptional control, and redox homeostasis under late stress response [74,75,76,77,78]. The MEgreenyellow module was enriched with genes such as *PHT*, *CPK*, *FLS2*, *galM*, and *P4HA*, indicating enhanced roles in nutrient transport, calcium-mediated signaling, receptor-like kinase pathways, and cell wall or metabolic remodeling during late-stage response [79,80,81,82,83].

Modules MEyellow and MEbrown were correlated with both early and late responses and enriched in genes regulating stress signaling, protein folding, and cell wall modification. Key hub genes at NaCl18 included *FMO*, *CYP450s* (*CYP90C1*, *CYP73A/84A/98A*), *HD-ZIP* transcription factors, *ZEP/ABA1*, *HSP20*, *PP2C*, and *PAO*, coordinating secondary metabolism, hormone signaling, carbohydrate metabolism, protein stabilization, and ROS homeostasis [72,84,85,86,87]. The concordance between hub-gene expression and metabolite accumulation, particularly phenylpropanoid derivatives and glutathione-related metabolites, underscores coordinated regulation of core stress-response pathways. Collectively, these results support a multi-layered tolerance strategy in *A. venetum*, with early signaling and osmotic adjustment transitioning into sustained metabolic, antioxidant, and structural acclimation under late stress responses.

Collectively, these results demonstrate that *A. venetum* employs a temporally coordinated, multi-layered salt-tolerance strategy, in which early signaling and osmotic adjustment transition into sustained metabolic, antioxidant, and structural acclimation under late stress responses. Importantly, the involvement of the identified phenylpropanoid metabolism, hormone signaling, and ROS-regulatory pathways suggests that the regulatory modules and candidate genes identified here may provide a valuable reference framework for dissecting and engineering salt-tolerance mechanisms in other agriculturally important crops.

## 4. Materials and Methods

### 4.1. Plant Material and Growth Conditions

Salt-tolerant *A. venetum* seedlings were obtained from the Qingdao Key Laboratory of Coastal Saline-alkali Land Resources Mining and Biological Breeding, China. Seeds were surface-sterilized, germinated, and grown in pots containing a peat:vermiculite substrate (1:1, *v*/*v*) under controlled conditions (25 ± 2 °C, 60–70% relative humidity, 16 h light/8 h dark). Uniform two-month-old plants were selected for stress treatments. Each treatment included three biological replicates, with each replicate consisting of pooled leaves from three plants.

### 4.2. Experimental Design and Salt Treatments

Salt stress was imposed using 200 mM NaCl, a concentration commonly applied in salt-tolerance studies. Five sample groups were established: CK0 (control, day 0), CK7 (control, day 7), CK18 (control, day 18), NaCl7 (200 mM NaCl, day 7), and NaCl18 (200 mM NaCl, day 18). NaCl treatment began at day 0 and was applied only once via irrigation, while control plants received NaCl-free irrigation. During treatment, pots were placed in trays, and water or saline solution was added to the trays to allow bottom-up soil absorption until the entire soil profile, including the surface layer, was fully moistened. Leaf samples were collected at 7 and 18 days to represent early and late stress responses. The experiment followed a randomized complete block design with three biological replicates per group.

### 4.3. Sampling and Tissue Processing

At each time point (day 7 and 18), fully expanded mature leaves were harvested from the middle canopy layer of plants, flash-frozen in liquid nitrogen within 30 s, and stored at −80 °C. For each biological replicate, tissues from three plants were pooled. Aliquots from the same pooled leaf samples were used for physiological and biochemical indicator measurements as well as for transcriptomic and metabolomic analyses, enabling direct integration of DEGs and DAMs in a time-resolved multi-omics framework.

### 4.4. Physiological and Biochemical Analyses

For sodium (Na^+^) determination, samples were oven-dried at 65 °C to a constant weight and ground. Then, 100 mg aliquots were digested overnight in HClO_4_:HNO_3_ (1:2 *v*/*v*) following a published protocol with modifications [88]. Na^+^ concentrations were quantified using an atomic absorption spectrophotometer (PerkinElmer Model 360, PerkinElmer Inc., Shanghai, China) and calculated against standard calibration curves. For antioxidant enzyme assays, approximately 0.5 g fresh leaf tissues per replicate were homogenized in 5ml extraction buffer (0.1 mM EDTA, 0.1 M phosphate buffer, 1% PVP, pH 7.8) at 4 °C. Homogenates were centrifuged for 15 min (10,000× *g*, 4 °C), and supernatants were used for assays [89]. Catalase (CAT) activity was determined according to Beauchamp and Fridovich (1971), and sodium dismutase (SOD) activity following Beers and Sizer (1952) [90,91]. Malondialdehyde (MDA) content was measured as thiobarbituric acid-reactive substances after extraction with 10% TCA [89,92]. Total flavonoids were extracted with 80% methanol and quantified using a rutin standard curve as described by Sultana et al. (2009) with modifications [93,94]. Leaf pigments were extracted in 95% ethanol under dark conditions, and chlorophyll content was calculated following Lichtenthaler & Wellburn (1973) using absorbance at 663 and 646 nm [95].

$$Chlorophyll\;a\;content\;\left(mg/g\;FW\right)=\left(13.95\times A_{665}-6.88\times A_{699}\right)\frac{V}{W\times 1000}$$

$$Chlorophyll\;b\;content\;(mg/g\;FW)=(24.96\times A_{649}-7.32\times A_{665})\frac{V}{W\times 1000}$$

Total chlorophyll content=Chlorophyll a content+Chlorophyll b content

### 4.5. Transcriptome (RNA-Seq) Analysis

Total RNA was extracted using a plant RNA isolation kit optimized for polyphenol-rich tissues. RNA quality was assessed using a Bioanalyzer (RIN ≥ 7) and quantified with Qubit. Poly(A)-enriched mRNA libraries were constructed and sequenced on an Illumina NovaSeq platform (PE150) by Majorbio Bio-Pharm Technology Co., Ltd. (Shanghai, China; https://www.majorbio.com, last accessed on 12 December 2025). Quality control was performed using FastQC, and low-quality reads were trimmed with Trimmomatic [96]. Due to the absence of a reference genome, de novo transcriptome assembly was conducted using Trinity [97]. Gene expression levels were quantified with RSEM [98]. Differentially expressed genes (DEGs) were identified using DESeq2 on the Majorbio Cloud Platform, with FDR < 0.05 and |log2FC| ≥ 1 as significance thresholds. Functional enrichment for GO and KEGG pathways was performed using the Majorbio cloud platform (https://cloud.majorbio.com/, last accessed on 12 December 2025). Weighted gene co-expression network analysis was carried out using the Majorbio Cloud Platform (https://cloud.majorbio.com/, last accessed on 12 December 2025) to detect modules associated with NaCl stress at 7 and 18 days. Module eigengenes were extracted and correlated with selected metabolite profiles using all biological samples across treatments and time points (*n* = 15; 3 replicates per group) on the Major cloud platform (https://cloud.majorbio.com/, last accessed on 12 December 2025), with Pearson correlation used to calculate correlation coefficients and *p*-values (|r| ≥ 0.7, *p* < 0.05). For genes represented by multiple transcripts, expression values were summarized at the gene level by selecting the transcript with the highest mean expression across biological replicates. Transcript-level information is provided in Table S1.

### 4.6. Metabolomics Analysis

Metabolite extraction followed MetWare (Wuhan, China; https://www.metwarebio.com, last accessed on 12 December 2025) standardized protocols. Freeze-dried tissues were ground and extracted using 70% methanol with internal standards. Extracts were filtered and analyzed on a UPLC-ESI-MS/MS system operated by MetWare (https://www.metwarebio.com, last accessed on 12 December 2025), covering both positive and negative ionization modes. QC samples were injected periodically to monitor signal stability. MetWare cloud platform (https://cloud.metware.cn/#/home, last accessed on 12 December 2025) was used for peak detection, alignment, quantification, metabolite annotation, and statistical analyses. Differentially accumulated metabolites (DAMs) were defined using fold-change thresholds and adjusted *p*-values (Benjamini-Hochberg correction). PCA and heatmaps were generated using platform tools and R [99].

### 4.7. Integrated Transcriptome-Metabolome Analysis

Joint analysis was conducted on the Majorbio (https://cloud.majorbio.com/, last accessed on 12 December 2025). DEGs and DAMs were mapped to shared KEGG pathways and correlation matrices. Highly significant hub genes were visualized using Cytoscape v3.9.1. Heatmaps and correlation networks from integrated omics data were generated using the Majorbio cloud platform (https://cloud.majorbio.com/, last accessed on 12 December 2025).

### 4.8. Statistical Analyses

Statistical summaries were generated in Excel and R v4.2 [99]. Differences among treatments were evaluated by one-way ANOVA followed by Tukey’s multiple comparison test (*p* < 0.05) in R v4.2 [99]. Omics-specific significance thresholds followed DESeq2 (transcriptomics FDR) and metabolomics adjusted *p*-values. Multivariate analyses (PCA, clustering heatmaps) were implemented using standard R v4.2 packages and Majorbio cloud toolkits platform (https://cloud.majorbio.com/, last accessed on 12 December 2025).

## 5. Conclusions

*A. venetum* exhibited a phase-specific response to NaCl stress, characterized by early (7-day) activation of hormone signaling (ABA, auxin, zeatin) and MAPK pathways, followed by late (18-day) enhancement of phenylpropanoid and glutathione metabolism, secondary metabolite accumulation, and amino acid-mediated osmotic regulation. These molecular responses were accompanied by coordinated physiological and biochemical adjustments, including reduced chlorophyll content, controlled Na+ accumulation, enhanced SOD and CAT activities, transitional MDA activity, and increased flavonoid levels. WGCNA resolved distinct regulatory modules underlying these responses. During the early response, genes including *PMA1/2*, *MAPKKK17/18*, *HSP20*, and *CYP98A* mediated rapid ion transport, stress signaling, protein stabilization, and phenylpropanoid activation. In the late response, genes such as *CYCD3*, *ARF*, *CPK*, and *FLS2* supported growth-stress coordination, hormone and calcium signaling, and metabolic remodeling. Genes expressed across both phases featured core regulators such as *CYP90C1* and *PP2C*, integrating hormone signaling, secondary metabolism, and ROS homeostasis. Together, these findings define a multi-layered, time-specific tolerance framework and highlight halophyte-derived candidate metabolites and genes that may be utilized to improve salt tolerance in agriculturally important crops.

## Data Availability

The original contributions presented in this study are included in the article and Supplementary Materials. Further inquiries can be directed to the corresponding author.

## Supplementary Materials

The following supporting information can be downloaded at: https://www.mdpi.com/article/10.3390/ijms27041917/s1.
