# Supplementary material for: Temporal Transcriptomic and Metabolomic Reprogramming Unveils a Two-Phase Salt Tolerance Mechanism in Apocynum venetum
Source: Int J Mol Sci. 2026 Feb 17;27(4):1917. doi: 10.3390/ijms27041917 (PMC12940728; doi:10.3390/ijms27041917)
Supplement: Supplementary file 1 [file ijms-27-01917-s001.zip › Figure S2.pdf]

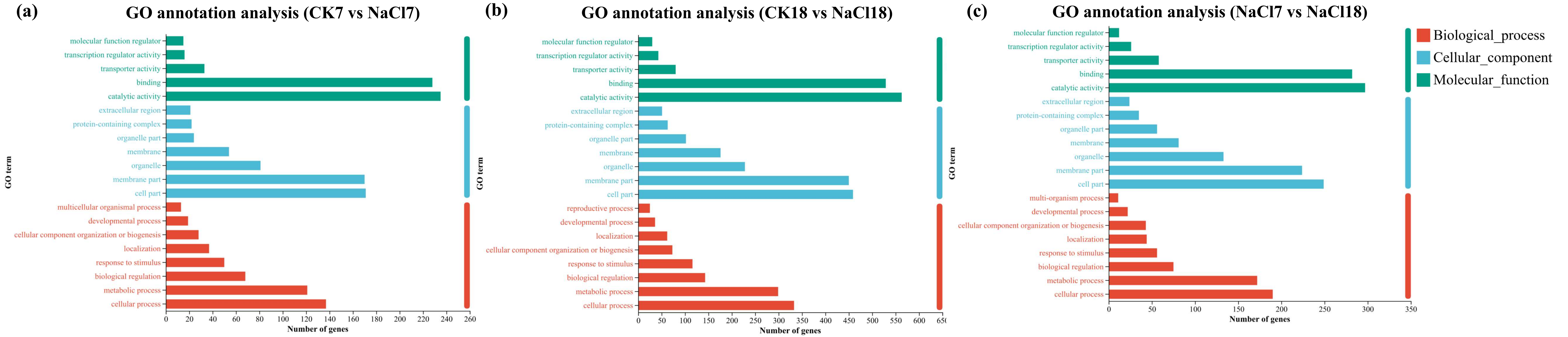

(a)
GO annotation analysis (CK7 vs NaCl7)
molecular function regulator
transcription regulator activity
transporter activity
binding
catalytic activity
extracellular region
protein-containing complex
organelle part
membrane
organelle
membrane part
cell part
multicellular organismal process
developmental process
cellular component organization or biogenesis
localization
response to stimulus
biological regulation
metabolic process
cellular process
GO term
0 20 40 60 80 100 120 140 160 180 200 220 240 260
Number of genes
(b)
GO annotation analysis (CK18 vs NaCl18)
molecular function regulator
transcription regulator activity
transporter activity
binding
catalytic activity
extracellular region
protein-containing complex
organelle part
membrane
organelle
membrane part
cell part
reproductive process
developmental process
localization
cellular component organization or biogenesis
response to stimulus
biological regulation
metabolic process
cellular process
GO term
0 50 100 150 200 250 300 350 400 450 500 550 600 650
Number of genes
(c)
GO annotation analysis (NaCl7 vs NaCl18)
molecular function regulator
transcription regulator activity
transporter activity
binding
catalytic activity
extracellular region
protein-containing complex
organelle part
membrane
organelle
membrane part
cell part
multi-organism process
developmental process
cellular component organization or biogenesis
localization
response to stimulus
biological regulation
metabolic process
cellular process
GO term
0 50 100 150 200 250 300 350
Number of genes
Biological_process
Cellular_component
Molecular_function
