# Supplementary material for: Temporal Transcriptomic and Metabolomic Reprogramming Unveils a Two-Phase Salt Tolerance Mechanism in Apocynum venetum
Source: Int J Mol Sci. 2026 Feb 17;27(4):1917. doi: 10.3390/ijms27041917 (PMC12940728; doi:10.3390/ijms27041917)
Supplement: Supplementary file 1 [file ijms-27-01917-s001.zip › Figure S3.pdf]

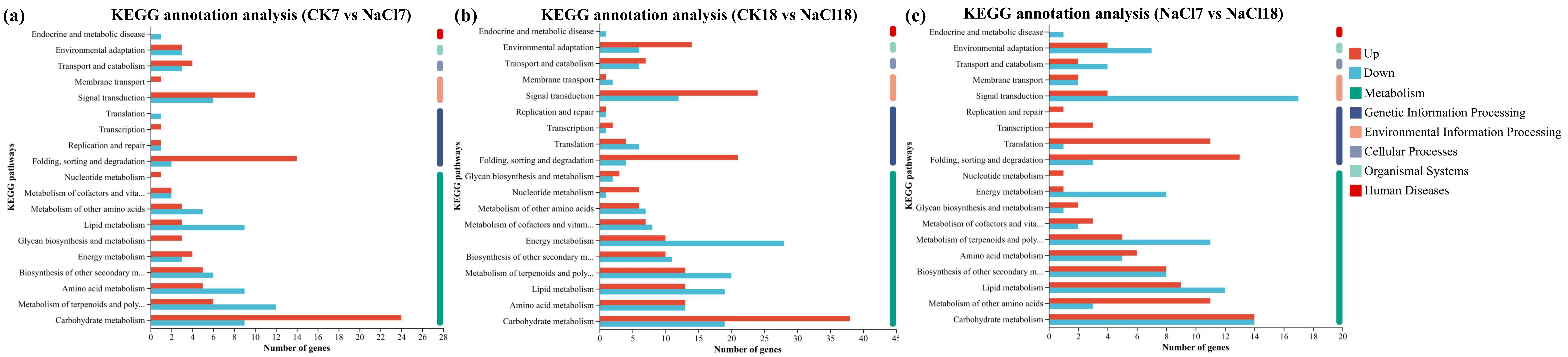

(a)
KEGG annotation analysis (CK7 vs NaCl7)
Endocrine and metabolic disease
Environmental adaptation
Transport and catabolism
Membrane transport
Signal transduction
Translation
Transcription
Replication and repair
Folding, sorting and degradation
Nucleotide metabolism
Metabolism of cofactors and vita...
Metabolism of other amino acids
Lipid metabolism
Glycan biosynthesis and metabolism
Energy metabolism
Biosynthesis of other secondary m...
Amino acid metabolism
Metabolism of terpenoids and poly...
Carbohydrate metabolism
KEGG pathways
Number of genes
0 2 4 6 8 10 12 14 16 18 20 22 24 26 28
(b)
KEGG annotation analysis (CK18 vs NaCl18)
Endocrine and metabolic disease
Environmental adaptation
Transport and catabolism
Membrane transport
Signal transduction
Replication and repair
Transcription
Translation
Folding, sorting and degradation
Glycan biosynthesis and metabolism
Nucleotide metabolism
Metabolism of other amino acids
Metabolism of cofactors and vitam...
Energy metabolism
Biosynthesis of other secondary m...
Metabolism of terpenoids and poly...
Lipid metabolism
Amino acid metabolism
Carbohydrate metabolism
KEGG pathways
Number of genes
0 5 10 15 20 25 30 35 40 45
(c)
KEGG annotation analysis (NaCl7 vs NaCl18)
Endocrine and metabolic disease
Environmental adaptation
Transport and catabolism
Membrane transport
Signal transduction
Replication and repair
Transcription
Translation
Folding, sorting and degradation
Nucleotide metabolism
Energy metabolism
Glycan biosynthesis and metabolism
Metabolism of cofactors and vita...
Metabolism of terpenoids and poly...
Amino acid metabolism
Biosynthesis of other secondary m...
Lipid metabolism
Metabolism of other amino acids
Carbohydrate metabolism
KEGG pathways
Number of genes
0 2 4 6 8 10 12 14 16 18 20
Up
Down
Metabolism
Genetic Information Processing
Environmental Information Processing
Cellular Processes
Organismal Systems
Human Diseases
