# Supplementary material for: Temporal Transcriptomic and Metabolomic Reprogramming Unveils a Two-Phase Salt Tolerance Mechanism in Apocynum venetum
Source: Int J Mol Sci. 2026 Feb 17;27(4):1917. doi: 10.3390/ijms27041917 (PMC12940728; doi:10.3390/ijms27041917)
Supplement: Supplementary file 1 [file ijms-27-01917-s001.zip › Figure S4.pdf]

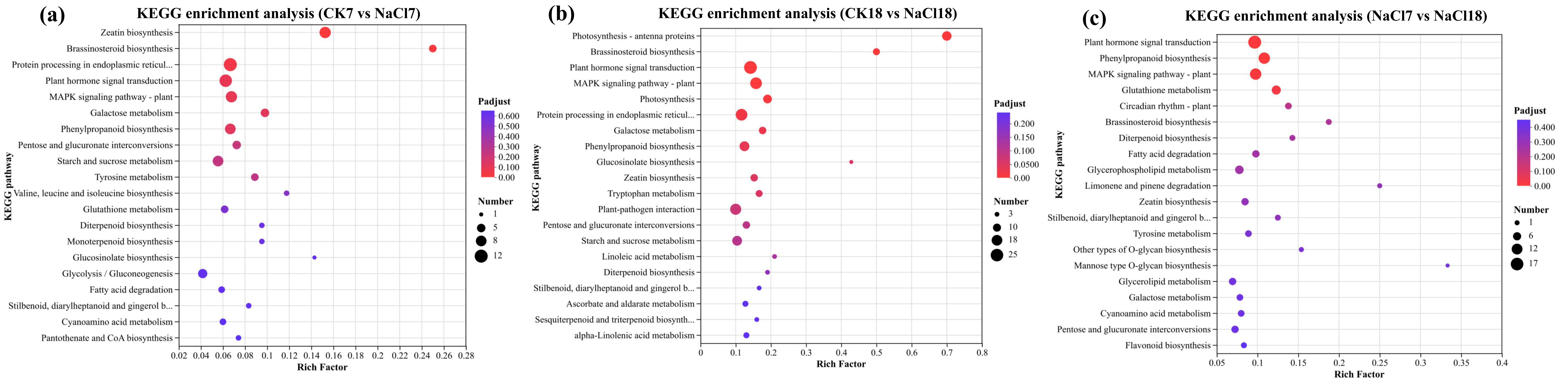

(a)
KEGG enrichment analysis (CK7 vs NaCl7)
Zeatin biosynthesis
Brassinosteroid biosynthesis
Protein processing in endoplasmic reticul...
Plant hormone signal transduction
MAPK signaling pathway - plant
Galactose metabolism
Phenylpropanoid biosynthesis
Pentose and glucuronate interconversions
Starch and sucrose metabolism
Tyrosine metabolism
Valine, leucine and isoleucine biosynthesis
Glutathione metabolism
Diterpenoid biosynthesis
Monoterpenoid biosynthesis
Glucosinolate biosynthesis
Glycolysis / Gluconeogenesis
Fatty acid degradation
Stilbenoid, diarylheptanoid and gingerol b...
Cyanoamino acid metabolism
Pantothenate and CoA biosynthesis
KEGG pathway
Rich Factor
0.02 0.04 0.06 0.08 0.1 0.12 0.14 0.16 0.18 0.2 0.22 0.24 0.26 0.28
Padjust
0.600 0.500 0.400 0.300 0.200 0.100 0.00
Number
1 5 8 12
(b)
KEGG enrichment analysis (CK18 vs NaCl18)
Photosynthesis - antenna proteins
Brassinosteroid biosynthesis
Plant hormone signal transduction
MAPK signaling pathway - plant
Photosynthesis
Protein processing in endoplasmic reticul...
Galactose metabolism
Phenylpropanoid biosynthesis
Glucosinolate biosynthesis
Zeatin biosynthesis
Tryptophan metabolism
Plant-pathogen interaction
Pentose and glucuronate interconversions
Starch and sucrose metabolism
Linoleic acid metabolism
Diterpenoid biosynthesis
Stilbenoid, diarylheptanoid and gingerol b...
Ascorbate and aldarate metabolism
Sesquiterpenoid and triterpenoid biosynth...
alpha-Linolenic acid metabolism
KEGG pathway
Rich Factor
0 0.1 0.2 0.3 0.4 0.5 0.6 0.7 0.8
Padjust
0.200 0.150 0.100 0.0500 0.00
Number
3 10 18 25
(c)
KEGG enrichment analysis (NaCl7 vs NaCl18)
Plant hormone signal transduction
Phenylpropanoid biosynthesis
MAPK signaling pathway - plant
Glutathione metabolism
Circadian rhythm - plant
Brassinosteroid biosynthesis
Diterpenoid biosynthesis
Fatty acid degradation
Glycerophospholipid metabolism
Limonene and pinene degradation
Zeatin biosynthesis
Stilbenoid, diarylheptanoid and gingerol b...
Tyrosine metabolism
Other types of O-glycan biosynthesis
Mannose type O-glycan biosynthesis
Glycerolipid metabolism
Galactose metabolism
Cyanoamino acid metabolism
Pentose and glucuronate interconversions
Flavonoid biosynthesis
KEGG pathway
Rich Factor
0.05 0.1 0.15 0.2 0.25 0.3 0.35 0.4
Padjust
0.400 0.300 0.200 0.100 0.00
Number
1 6 12 17
