# Supplementary material for: Temporal Transcriptomic and Metabolomic Reprogramming Unveils a Two-Phase Salt Tolerance Mechanism in Apocynum venetum
Source: Int J Mol Sci. 2026 Feb 17;27(4):1917. doi: 10.3390/ijms27041917 (PMC12940728; doi:10.3390/ijms27041917)
Supplement: Supplementary file 1 [file ijms-27-01917-s001.zip › Figure S5.pdf]

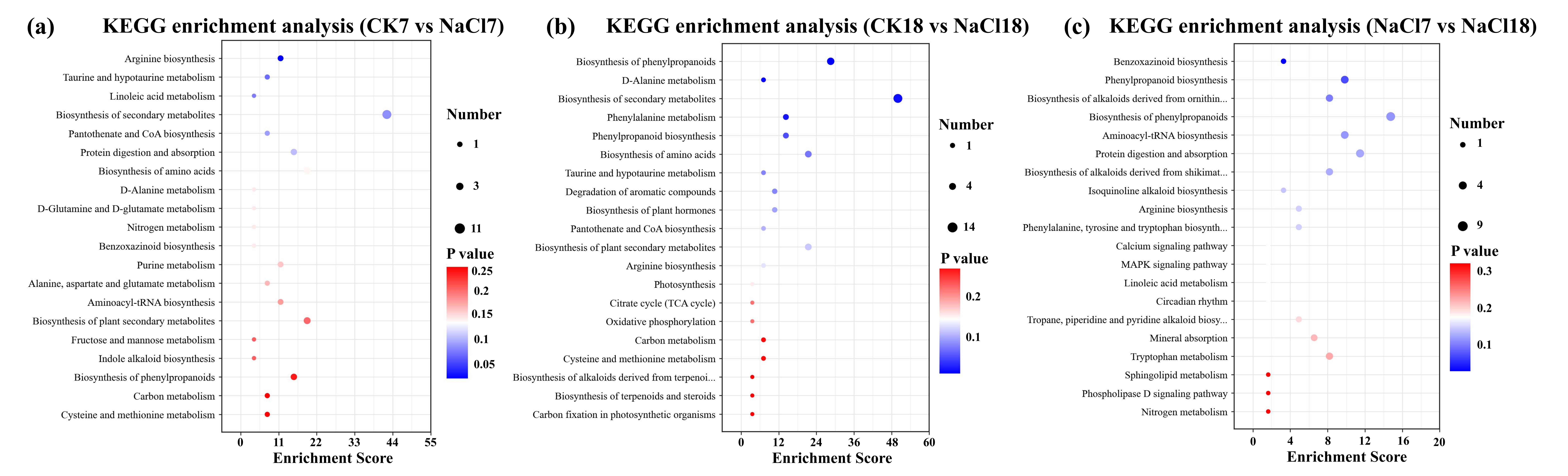

(a)
KEGG enrichment analysis (CK7 vs NaCl7)
Arginine biosynthesis
Taurine and hypotaurine metabolism
Linoleic acid metabolism
Biosynthesis of secondary metabolites
Pantothenate and CoA biosynthesis
Protein digestion and absorption
Biosynthesis of amino acids
D-Alanine metabolism
D-Glutamine and D-glutamate metabolism
Nitrogen metabolism
Benzoxazinoid biosynthesis
Purine metabolism
Alanine, aspartate and glutamate metabolism
Aminoacyl-tRNA biosynthesis
Biosynthesis of plant secondary metabolites
Fructose and mannose metabolism
Indole alkaloid biosynthesis
Biosynthesis of phenylpropanoids
Carbon metabolism
Cysteine and methionine metabolism
0 11 22 33 44 55
Enrichment Score
Number
1
3
11
P value
0.25
0.2
0.15
0.1
0.05
(b)
KEGG enrichment analysis (CK18 vs NaCl18)
Biosynthesis of phenylpropanoids
D-Alanine metabolism
Biosynthesis of secondary metabolites
Phenylalanine metabolism
Phenylpropanoid biosynthesis
Biosynthesis of amino acids
Taurine and hypotaurine metabolism
Degradation of aromatic compounds
Biosynthesis of plant hormones
Pantothenate and CoA biosynthesis
Biosynthesis of plant secondary metabolites
Arginine biosynthesis
Photosynthesis
Citrate cycle (TCA cycle)
Oxidative phosphorylation
Carbon metabolism
Cysteine and methionine metabolism
Biosynthesis of alkaloids derived from terpenoi...
Biosynthesis of terpenoids and steroids
Carbon fixation in photosynthetic organisms
0 12 24 36 48 60
Enrichment Score
Number
1
4
14
P value
0.2
0.1
(c)
KEGG enrichment analysis (NaCl7 vs NaCl18)
Benzoxazinoid biosynthesis
Phenylpropanoid biosynthesis
Biosynthesis of alkaloids derived from ornithin...
Biosynthesis of phenylpropanoids
Aminoacyl-tRNA biosynthesis
Protein digestion and absorption
Biosynthesis of alkaloids derived from shikimat...
Isoquinoline alkaloid biosynthesis
Arginine biosynthesis
Phenylalanine, tyrosine and tryptophan biosynth...
Calcium signaling pathway
MAPK signaling pathway
Linoleic acid metabolism
Circadian rhythm
Tropane, piperidine and pyridine alkaloid biosy...
Mineral absorption
Tryptophan metabolism
Sphingolipid metabolism
Phospholipase D signaling pathway
Nitrogen metabolism
0 4 8 12 16 20
Enrichment Score
Number
1
4
9
P value
0.3
0.2
0.1
