# Supplementary figures and images for: Temporal Transcriptomic and Metabolomic Reprogramming Unveils a Two-Phase Salt Tolerance Mechanism in Apocynum venetum
Source: Int J Mol Sci. 2026 Feb 17;27(4):1917. doi: 10.3390/ijms27041917 (PMC12940728; doi:10.3390/ijms27041917)

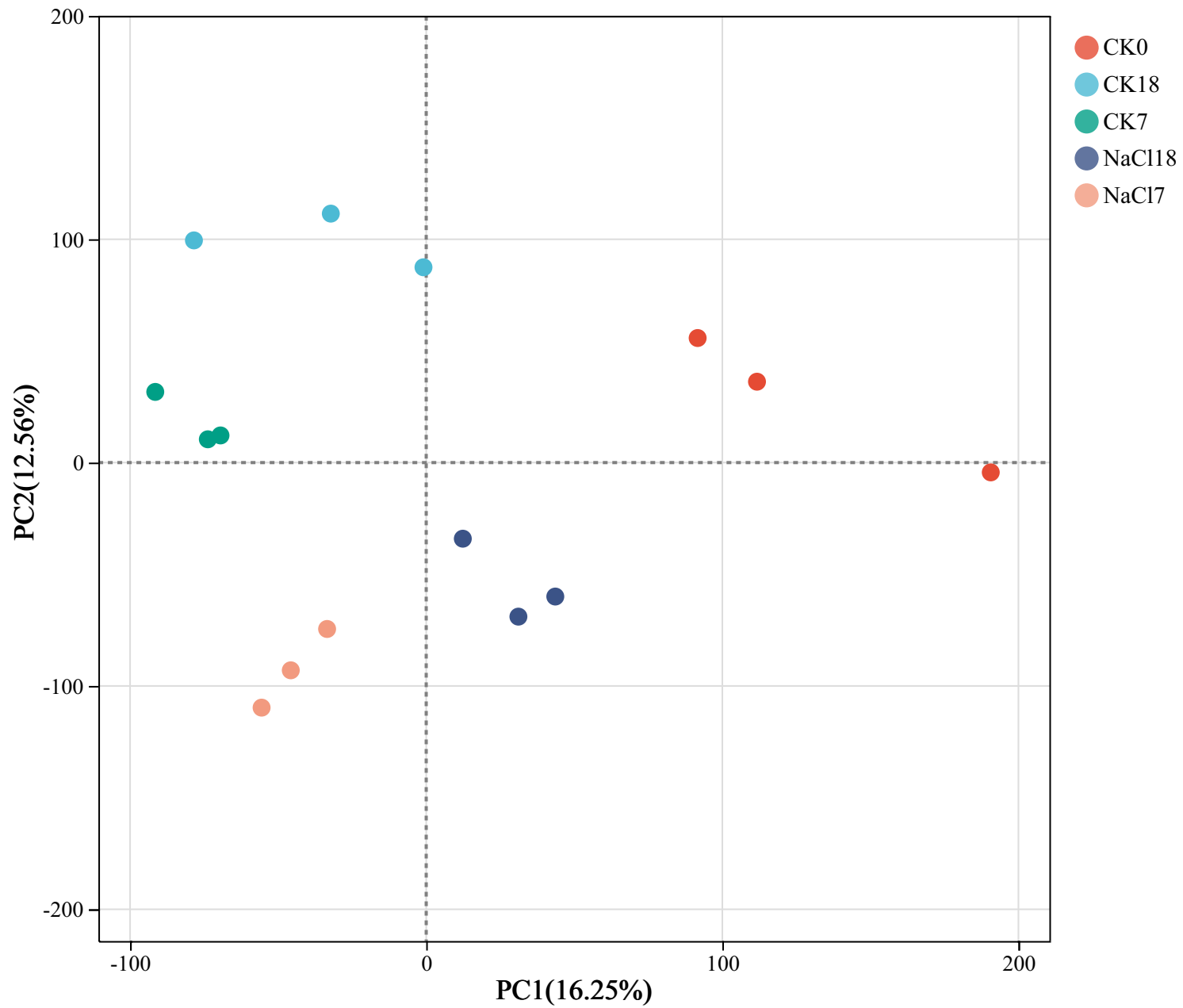

Supplement: Supplementary file 1 [file ijms-27-01917-s001.zip › Figure S1.pdf]
